# Pulmonary arteriovenous malformations with suspected infiltrative disease: A case report from a peripheral hospital

**DOI:** 10.21542/gcsp.2025.1

**Published:** 2025-02-28

**Authors:** Nabila Azka Namirah, Naufal Fakhri Nugraha, Zahra Nadiah, Zahran Lazuardi Haryawan, Abednego Panggabean

**Affiliations:** 1 Pameungpeuk Regional General Hospital, Garut, West Java, Indonesia; 2 Universitas Padjadjaran Hospital, Sumedang, West Java, Indonesia; 3 Universitas Padjadjaran, Sumedang, West Java, Indonesia; 4 Pameungpeuk Regional General Hospital, Garut, West Java, Indonesia

## Abstract

Pulmonary arteriovenous malformations (PAVMs) is a rare condition involving an abnormal connection of the pulmonary vasculature between the artery and vein, bypassing the capillary structure and causing a right-to-left shunt (RLS) of blood flow. This case report describes a 17-year-old female patient presenting with recurrent nose and tongue bleeding. Cyanosis and clubbing of the fingers were observed, along with visible telangiectasis on the skin and mucosal surfaces. Hereditary hemorrhagic telangiectasis (HHT) was diagnosed based on the Curaçao criteria. The RLS manifestations suggested a high probability of PAVM, confirmed by an agitated saline test showing a positive bubble appearance after four beats from the pulmonary vein to the left ventricle. An increased intraventricular wall diameter (19 mm) with a granular sparkling appearance indicated potential cardiac amyloidosis (CA). Speckle tracking echocardiography (STE) revealed a ’cherry-like’ appearance in a ’bull’s eye’ pattern. This case illustrates a diagnostic approach for PAVM in an adolescent HHT patient with suspected CA in a rural setting using limited resources.

## Background

Pulmonary arteriovenous malformations (PAVMs) is a rare condition involving an abnormal connection of the pulmonary vasculature between the artery and vein, bypassing the capillary structure and causing a right-to-left shunt (RLS) of blood flow^1,2^. PAVMs are associated with hereditary hemorrhagic telangiectasis (HHT) in the majority of cases^1–3^. However, PAVMs can be acquired in approximately 20% of cases. Acquired PAVMs have been associated with various underlying pathological conditions, including trauma, hepatic cirrhosis, infections, amyloidosis, metastatic cancer, lung parenchymal cysts, cardiothoracic surgery, mitral stenosis, and, in rare cases, constrictive pericarditis^3^.

Although PAVMs appear asymptomatic at birth, right-to-left shunt manifestation can develop over the years as dyspnea, fatigue, cyanosis, or hypoxemia, depending on the degree of right-to-left shunt^1,2^. Transthoracic contrast echocardiography (TTCE) is the recommended tool for screening PAVMs^1^. Screening is recommended in all adults with HHT even if there was no evidence of PAVMs in a previous examination. This is due to the possible development of PAVMs from childhood into adulthood^1^. PAVMs are confirmed *via* a CT scan in both child and adult patients after they are diagnosed with HHT^1^. This study describes our diagnostic approach for detecting PAVMs in patients with HHT at a rural hospital. We discuss the effective treatment strategies implemented and report our echocardiographic findings that suggested the presence of a rare cardiac infiltrative disease.

## Case presentation

A 17-year-old girl presented to a peripheral hospital with recurrent epistaxis and tongue bleeding that had become more frequent in the past month. She also felt fatigue and malaise, sometimes accompanied with chest pain. At first glance, the patient apperared to be cyanotic.

**Figure 1.**
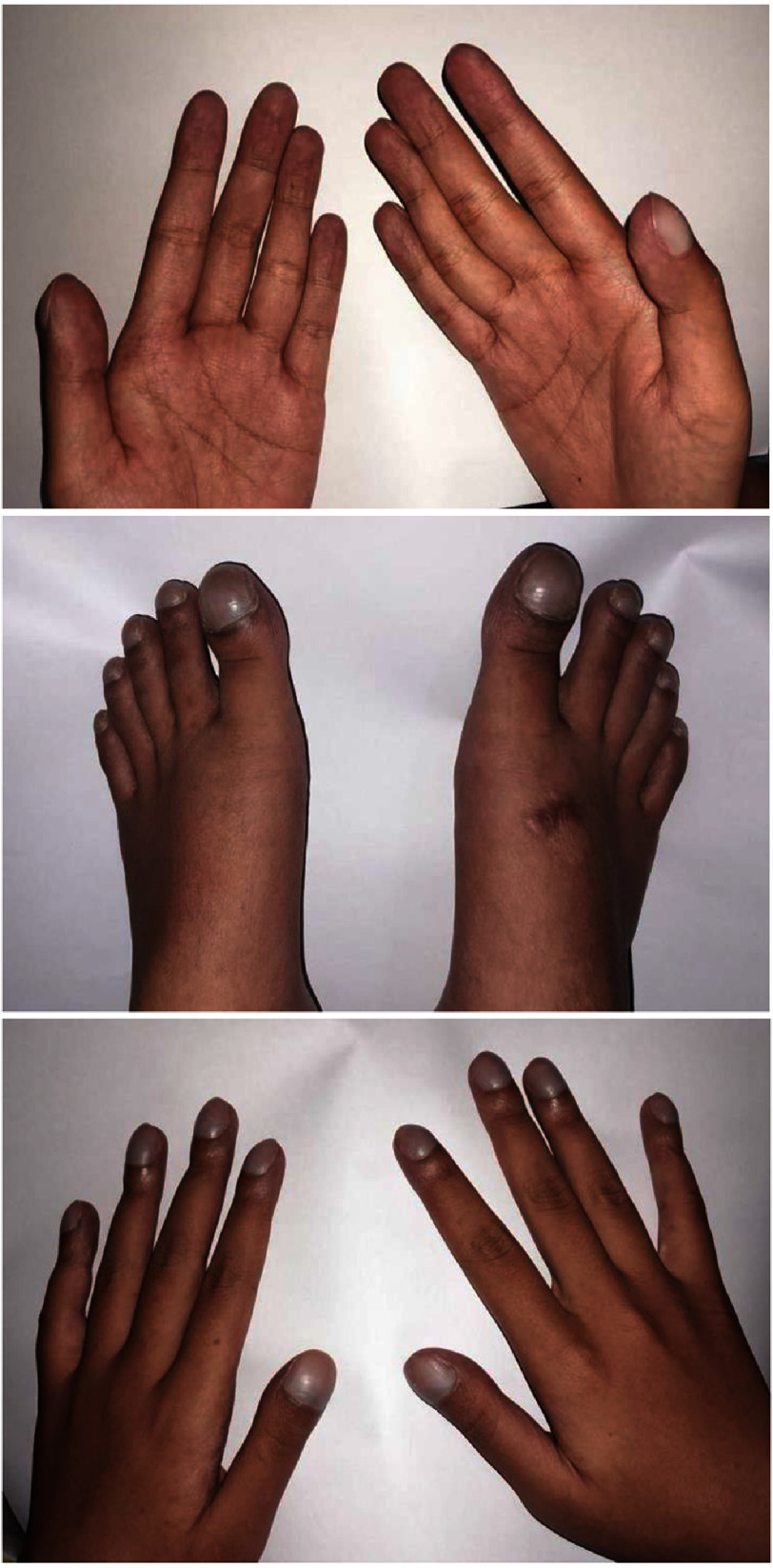
Cyanosis and clubbing fingers of the patient.

Blood pressure was elevated (170/90 mmHg) and heart rate was 108/min. Oxygen saturation was low at 84% without oxygen supplementation. Physical examination revealed clubbing fingers and toes (Figure 1) with telangiectasia on the upper extremities and face. Examination of the oropharyngeal mucosa revealed petechiae and the liver was palpably enlarged. Neither the patient nor family members had reported similar symptoms previously, nor was there any history of congenital heart disease in patient or family.

**Figure 2.**
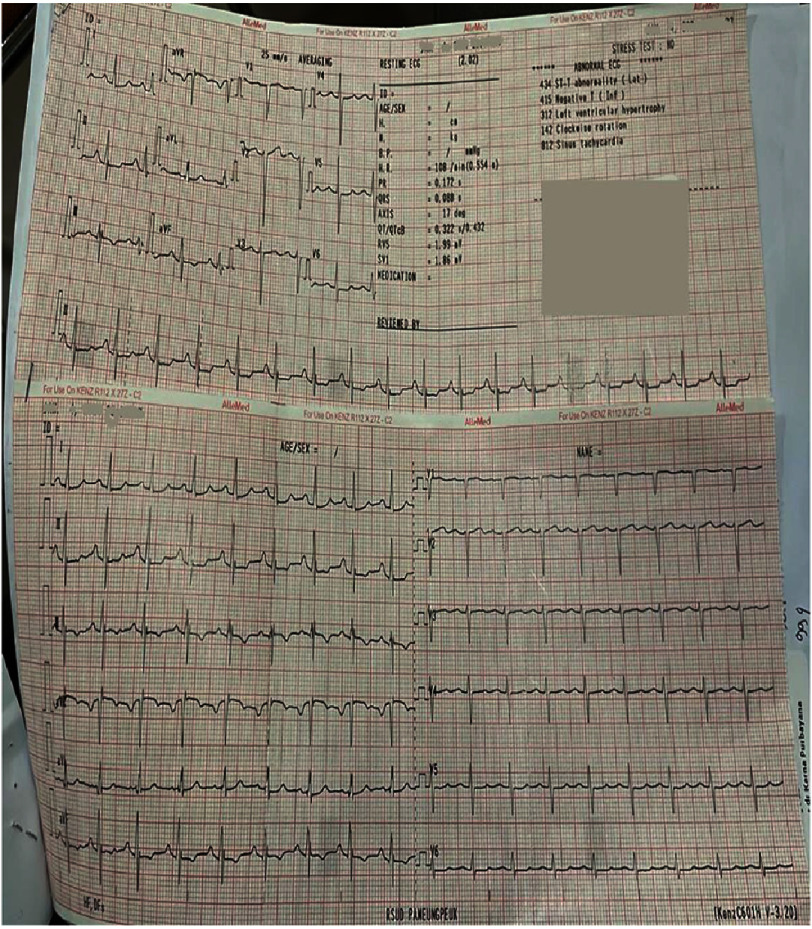
Electrocardiography results. Left ventricular hypertrophy based on Framingham with SV1-V3 > 25 mm.

Initial laboratory work-up showed high levels of hemoglobin (Hb): 18.4 d/dL, hematocrit (Hct) 62.6%, with increased erythrocyte 9.49 10^3^/μL. There was a low level of leukocyte 3.97 10^3^/μL, and normal platelet count of 269 10^3^/μL. Sinus tachycardia and left ventricle hypertrophy (LVH) were present in electrocardiography (ECG) (Figure 2). Chest radiography demonstrated cardiomegaly with a cardiothoracic ratio (CTR) greater than 50%.

**Figure 3.**
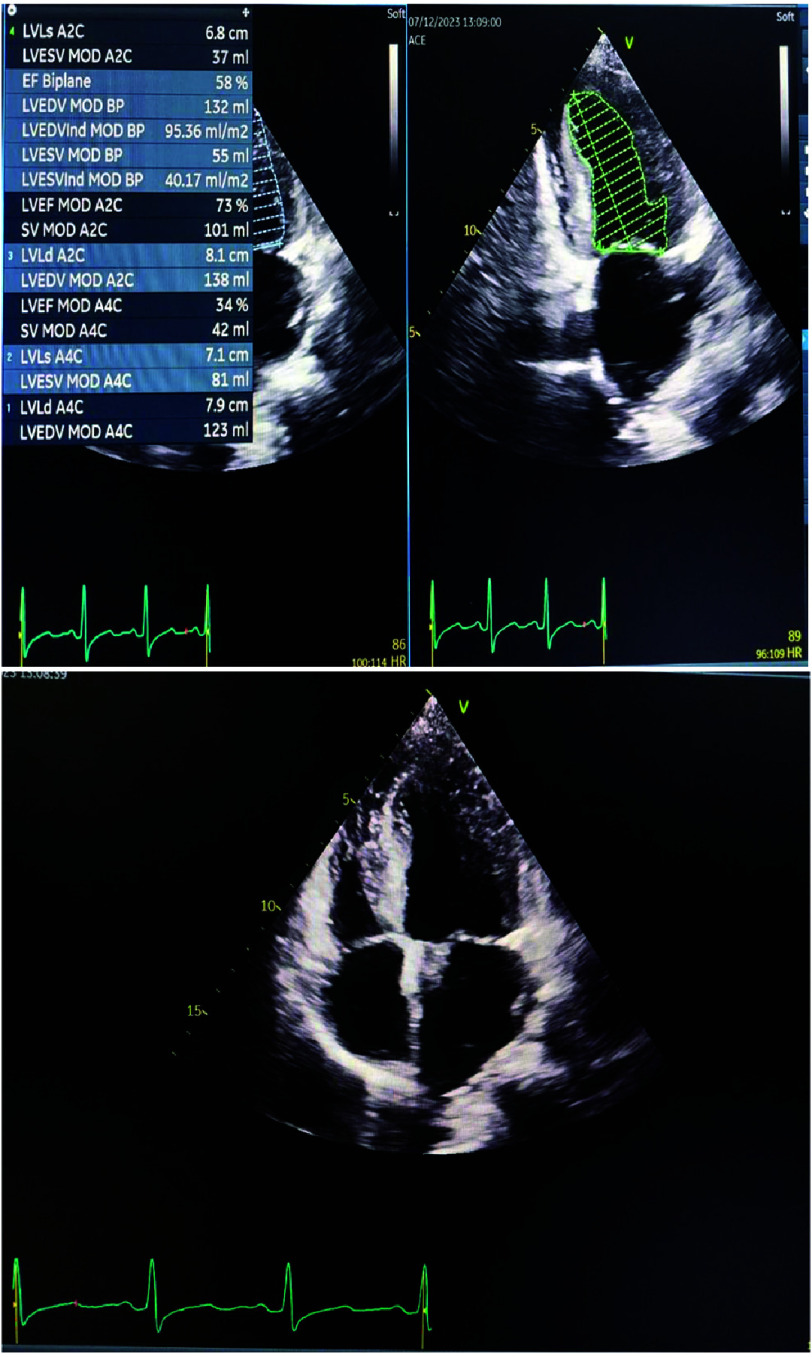
Apical two-chamber and four-chamber echocardiography. The ejection fraction (EF) was calculated in biplane mode (result 58%). Granular-sparkling appearance was evident in both the apical two-chamber and four-chamber echocardiographic views.

**Figure 4.**
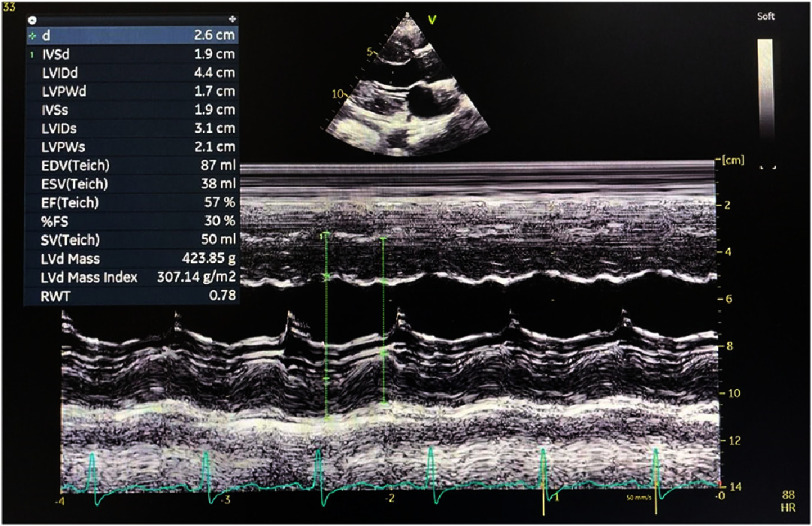
M-mode echocardiography. Intraventricular septal diameter was 1.9 cm.

Echocardiography was performed to assess cardiac function and identify any structural defects that could explain the patient’s right-to-left shunt. Ventricular ejection fraction (EF) was normal (58%) (Figure 3). However, apical four-chamber echocardiographic view revealed increased myocardial thickness with a granular-sparkling appearance. Intraventricular septal diameter (IVSd) was larger than normal at 19 mm (Figure 4).

**Figure 5.**
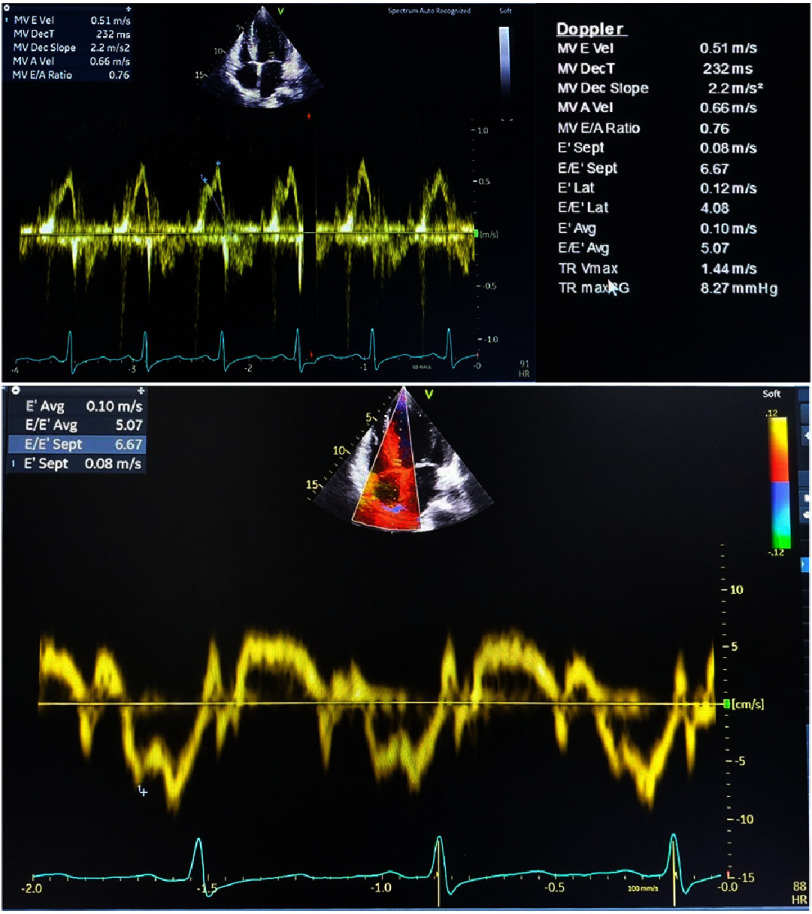
Doppler echocardiography. The E-wave represents early diastolic passive filling of the left ventricle, while the A-wave represents late diastolic blood flow generated by atrial contraction. In this patient, the TR Vmax was 1.44 m/s.

Doppler echocardiography allowed us to calculate mitral E-wave velocity (MV E Vel) 0.51 m/s, mitral A-wave velocity (MV A Vel) 0.66 m/s, ratio of the early E-wave to late A-wave ventricular filling velocity (MV E/A ratio) 0.76, lateral mitral annulus velocity (E’ lat) 0.12 ml, septal mitral annulus mitral velocity (E’ sept) 0.08 m/s with average E’ (E’ avg) 0.10 m/s, E/E’ avg 5.07, and tricuspid regurgitation velocity (TR Vmax) 1.44 m/s. To complete the assessment of diastolic function, left atrium end systolic velocity index (LA vol index) was investigated and the result was 59.04 ml/m2 (Figure 5).

**Figure 6.**
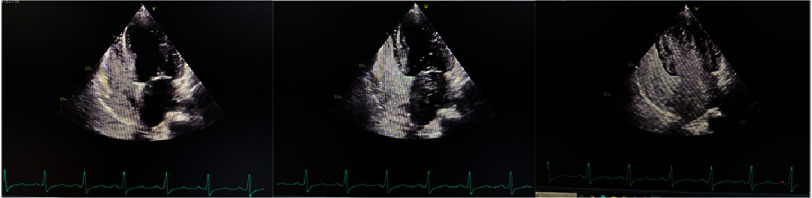
Agitated saline test. (A) Bubbles are visible at right atrium and right ventricle. (B) Bubbles are visible in left ventricle from pulmonary vein after 4 beats. (C) Positive bubbles appear in left ventricle.

An agitated saline test was performed to see any possible vascular malformation that could have caused RLS manifestation. After 1 beat, bubbles enter the right atrium. In 4 to 5 beats bubbles started entering left ventricle from pulmonary vein and after 5 beats, the bubbles clearly visualized in left ventricle, suggesting PAVM (Figure 6 & Video S1).

**Figure 7.**
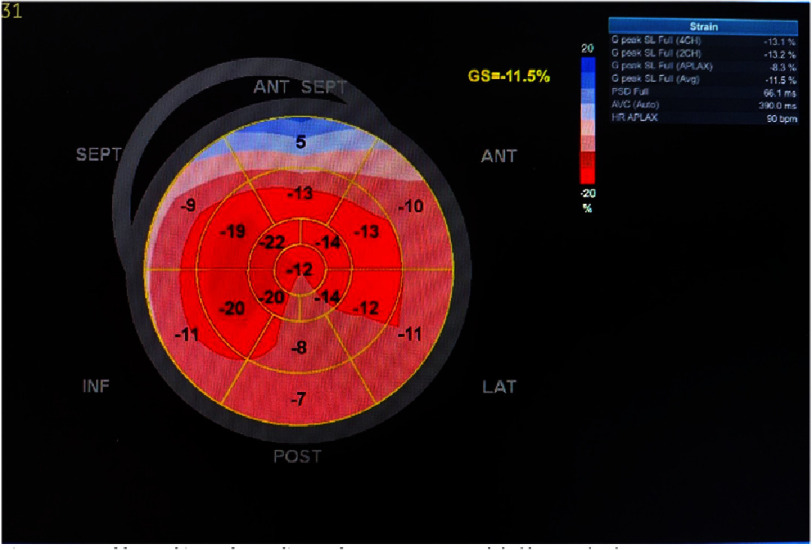
Speckle-tracking echocardiography (STE). Average global longitudinal strain (GLS) was -11.5%.

The increased interventricular septal thickness in diastole (IVSd) with a granular-sparkling appearance suggested cardiac infiltrative disease. Speckle tracking echocardiography demonstrated reduced global longitudinal strain of −11.5% in the left ventricle, with an apical sparing (cherry-on-top) pattern (Figure 7). To differentiate from other causes of left ventricular hypertrophy, we calculated several diagnostic parameters: relative apical sparing (RELAPS), ejection fraction strain ratio (EFSR), and septal apical to base ratio (SAB). The values were 1.5, 5.04, and 1.9, respectively.

The presence of recurrent epistaxis and multiple telangiectases fulfill at least two diagnostic criteria of the Curaçao system for clinical diagnosis of HHT. The presence of PAVMs, confirmed by the positive contrast-enhanced transthoracic echocardiography (bubble study) and right-to-left shunt (RLS), manifesting as cyanosis, hypoxemia, and digital clubbing, provides additional diagnostic evidence supporting the diagnosis of HHT.

The echocardiographic findings of increased interventricular septal thickness, characteristic granular sparkling pattern, reduced global longitudinal strain, and the pathognomonic ’cherry on top’ sign on speckle-tracking analysis suggested cardiac amyloidosis as the underlying infiltrative cardiomyopathy. RELAPS and ESFR analysis strongly supported the suggestion of amyloidosis. While endomyocardial biopsy remains the gold standard for definitive diagnosis of cardiac amyloidosis, this procedure could not be performed due to limited institutional resources and facilities.

The patient was recommended for tertiary center referral for comprehensive evaluation and definitive management. However, due to socioeconomic constraints and geographical barriers to the referral center, the patient declined further specialized care. Conservative management was preferred, and symptomatic therapy was initiated with furosemide (20 mg daily), bisoprolol (2.5 mg daily), and acetaminophen (500 mg thrice daily). While symptomatic improvement was noted following supportive therapy, the patient’s clinical signs remained largely unchanged. After five months of outpatient follow-up, the patient ultimately consented to tertiary center referral.

## Discussion

PAVMs are vascular abnormalities between pulmonary arteries and veins, resulting in right-to-left shunting^1–3^. These vascular anomalies are predominantly (80%) congenital in nature and strongly associated with HHT^2,3^.

HHT, also known as Osler-Weber-Rendu syndrome, is an autosomal dominant disorder that results in abnormal development of artery-vein vasculature^4^. Arteriovenous malformations represent a major diagnostic feature of HHT and constitute one of the four Curaçao criteria for establishing a positive diagnosis (recurrent spontaneous epistaxis, visible telangiectasis, visceral arteriovenous malformation, and a first-degree relative with HHT)^3,4^.

PAVMs develop in up to 50% of HHT patients, with a higher prevalence in HHT type 1 (associated with ENG mutations) compared to HHT type 2 (ACVRL1 mutations)^1,4^. Though predominantly congenital, PAVMs may also develop secondary to pathological processes that affect angiogenic pathways including chest trauma, infectious diseases, post-cardiothoracic surgery complications, cirrhosis, mitral stenosis, metastatic cancer, and amyloidosis^1,3^.

The incidence of PAVMs in the general population is low^1,3^ but it occurs in 60% of cases of pediatric HHT. Approximately 30% of cases could not be detected at first screening in children with HHT, and the disorder developed over subsequent years^5^.

PAVMs have a wide spectrum of manifestations from asymptomatic to symptomatic clinical appearances that may be associated with the size of PAVMs, and exhibit right-to-left shunts of differing severity^1^. Increased degrees of RLS may contribute to a higher mix of deoxygenated blood, resulting in fatigue, hypoxemia, dyspnea, chest pain, cyanosis, and clubbing, through to possible complications such as cerebrovascular events, abscess, seizure, hemoptysis, and hematothorax^1,2^.

Although the severity of clinical manifestations typically correlates with the degree of right-to-left shunting, serious complications can still occur in PAVMs with feeding arteries less than three mm in diameter^1^. In this case, RLS manifestation was the primary reason we suspected PAVM in this patient. Additionally, we also met two of four Curaçao criteria for HHT (namely recurrent bleeding of nose and tongue, and visible telangiectasis).

Owing to its rarity, early recognition of congenital vascular malformations is challenging in peripheral healthcare settings, primarily due to limited awareness and diagnostic capabilities. We had to rely on a strong suggestion of PAVMs and the Curaçao criteria, plus a progresson of RLS over time.

Detection of PAVMs can make use of multiple diagnostic modalities, including pulse oximetry, chest radiography, transthoracic echocardiography, computed tomography (CT), and magnetic resonance imaging (MRI)^1,3,5^. Transthoracic contrast echocardiography (TTCE) is the screening test of choice for PAVM with sensitivity up to 98.6%, followed by CT scan to confirm the diagnosis for grade 2/3 TTCE shunt^1,2,5^.

Negative results in initial TTCE screening does not preclude the necessity for subsequent follow-up evaluations, as the natural progression of right-to-left shunts during childhood development may yield positive findings in later years^1^.

The gold standard of PAVM is CT scan to see the feeding artery and establish the diameter of PAVMs^1,3^. Moreover, TTCE can show the likelihood of PAVM from RLS grade and indicate the probability for embolization^6^. Echocardiography is a useful tool in screening for PAVM due to inherent advantages such as good sensitivity, low cost, non-invasive procedure and no radiation exposure^6^. In HHT patients, TTCE screening is suggested find to common PAVM features within the disease^1^.

Conversely, Lim et al. demonstrated that combining the agitated saline contrast test with graded transesophageal contrast echocardiography (TECE) provides an effective initial screening methodology for detecting RLS in patients without HHT^7^.

In our patient with clinically diagnosed HHT, we observed delayed contrast microbubble appearance in the left ventricle *via* the pulmonary vein after four cardiac cycles. Although computed tomography (CT), the gold standard for PAVM diagnosis, was not available at our facility, the high sensitivity of TTCE, reported at 98%, strengthens our presumptive diagnosis of PAVM in this peripheral hospital setting.

Our patient had normal ventricular EF, however, echocardiography showed increased IVSd with granular sparkling appearance - typically found in cardiac amyloidosis (CA)^8^. Other chracteristics that help to differentiate CA from other causes of cardiomyopathy include the increased IVSd ≥12 mm (with low or normal QRS voltage in ECG), visible slight pericardial effusion, biatrial wall enlargement, diastolic dysfunction, and cardiac valve enlargement^9^.

Cardiac amyloidosis, characterized by the pathological deposition of amyloid fibrils, represents a common etiology of restrictive cardiomyopathy (RCM). that can be specified within the heart, or as part of systemic amyloidosis^8,10^.

Left ventricular ejection fraction (LVEF) is typically preserved (HFpEF) until advanced stages of cardiac amyloidosis^9^. The American Society of Echocardiography recommends two initial approaches for diastolic function assessment in suspected restrictive cardiomyopathy (RCM): (1) reduced LVEF with myocardial disease, or (2) preserved LVEF with supportive clinical findings or two-dimensional echocardiographic data^8,11^.

Assessment with Doppler echocardiography shows mitral inflow and pulmonary vein flow, and speckle-tracking echocardiography (STE) can help to calculate cardiac strain^11^**.** LV diastolic function varies from grade 1 with normal LV filling pressure and affected relaxation to grade 2 in early stage CA^11^. Advanced diastolic dysfunction in late stage CA is related with poor outcomes due to the impaired relaxation with elevated LV filling pressure^11^.

A variety of clinical manifestations and imaging of CA can mimic some other cardiac illnesses such as hyperthropic cardiomyopathy, heart failure with preserved ejection fraction, and hypertensive heart disease, making definitive diagnosis more challenging^12^.

Speckle tracking echocardiography enables assessment of systolic parameters, including longitudinal, circumferential, and radial strain, which typically demonstrate abnormalities in cardiac amyloidosis^8^. When high LV filling pressure is detected, speckle-tracking echocardiography can help measure the decrease in global longitudinal strain (GLS)^8^. In CA, the decrease of apical longitudinal strain compared with other segments exhibits as ‘cherry on top’ using Bull’s eye pattern (Figure 7)^12^.

EFSR, SAB and RELAPS are established parameters for quantifying CA through STE assessment of LV longitudinal strain. These parameters effectively differentiate CA from other causes of left ventricular hypertrophy (LVH). The diagnostic thresholds have been established as follows: EFSR >4.1 (sensitivity 89.7%, specificity 91.7%), SAB >2.1 (sensitivity 65%, specificity 53%), and RELAPS ≥1.0 (sensitivity 62.5%, specificity 93.3%)^13^.

In their comprehensive review, Kiorsekeglou and colleagues synthesized findings from multiple studies to identify key echocardiographic parameters that raise clinical suspicion for cardiac amyloidosis (CA). These diagnostic indicators include: an apex-to-base left ventricular strain ratio exceeding 2.5; grade 2 or higher diastolic dysfunction; right ventricular apical sparing pattern with apical free wall strain greater than 27%; right atrial reservoir strain above 24% (as measured by three-dimensional speckle-tracking echocardiography); left atrial reservoir strain less than 29%; and increased ventricular wall thickness (posterior wall >13.6 mm and septal wall >16 mm)^12^.

Evaluation of our patient using Doppler echocardiography and speckle-tracking echocardiography (STE) demonstrated preserved left ventricular ejection fraction with grade 1 diastolic dysfunction. The observed reduction in global longitudinal strain and characteristic “cherry on top” pattern heightened clinical suspicion for RCM, specifically cardiac amyloidosis. Application of previously validated strain parameters yielded results significantly consistent with CA diagnosis. While PAVM remains the likely etiology of right-to-left shunting, our echocardiographic findings suggest concurrent early-stage CA development.

Both cardiac amyloidosis and HHT are rare diseases^1,14^. Both are challenging to diagnose due to varying clinical manifestations and the need for multimodal examinations^1,14,15^. Major et al. demonstrated that HHT diagnosis is frequently delayed due to several factors: the condition’s relative rarity, the asymptomatic nature of arteriovenous malformations in young patients, the delayed onset of characteristic clinical manifestations (including recurrent epistaxis and telangiectasia), and the tendency for patients to be evaluated for isolated systemic manifestations across various medical specialties^16^.

Delayed diagnosis is also a problem in cases of amyloidosis, which is also rare^17^. Beside the association with HHT pathophysiology, PAVM was documented as a rare complication that can manifest in amyloidosis and sarcoidosis^1,18,19^. Development of PAVM has been detected in a geriatric amyloidosis patient who presented with recurrent pneumonia that had been assessed *via* bronchoscopy with transbronchial biopsy and histopathology assessment after imaging reassessment in follow-up^14^.

With regards to infection, it remains unclear whether PAVM contributes to the higher incidence of severe infections in HHT patients. Recent research indicates that bacteria trapped in pulmonary capillaries are effectively cleared by marginated neutrophils, and bypassing this process could increase the risk of septic complications^20^. However, PAVMs reduce the lungs’ ability to filter blood, increasing the likelihood of brain ischemia caused by septic emboli or sterile emboli, which can lead to bacterial infection in the affected area^21^.

Previous studies have highlighted cases of HHT and amyloidosis in a geriatric patient^22^, but HHT with PAVM were also detected with pediatric sarcoidosis cases. This was hypothesized from the proinflammatory cytokine that caused vasodilatation which subsequently developed into sarcoidosis^18,19^. Although it remains unclear, the reported cases may contribute to our knowledge about the relation of PAVM in HTT and their relationship with amyloidosis.

## Conclusion

To the best of our knowledge, this represents the first documented case of a pediatric patient from a rural setting presenting with the concurrent findings of PAVM in the context of HHT and suspected cardiac amyloidosis. This case can open our minds to the possibility of a co-occurrence of CA and HTT, suggesting the need for further investigation into whether these conditions arise from distinct pathophysiological pathways or share a common underlying mechanism.

Since RCM in cardiac amyloidosis typically has a poor prognosis, prompt screening with multimodal imaging and biomarkers are important to achieve better outcomes in cardiac amyloidosis^9^. Untreated PAVM is a progressively life-threatening condition and can lead to ruptured PAVMs due to *e.g.*, worsening pulmonary hypertension^1^.

Other investigations need to be performed to confirm the diagnosis so the appropriate treatment can be performed. Early diagnosis can help the patient be more aware about the risk of disease progression and how it can cause mortality, especially in rural areas.

## What have we learned?

Diagnosing pulmonary arteriovenous malformations with suspected cardiac infiltrative diseases, such as cardiac amyloidosis, presents significant challenges, particularly in peripheral healthcare settings with limited resources. This case underscores the necessity of a structured approach to echocardiographic assessment, incorporating contrast-enhanced imaging and speckle-tracking echocardiography to facilitate accurate diagnosis.

Given the potential for PAVM progression and its associated complications, early identification and systematic screening of individuals suspected of having hereditary hemorrhagic telangiectasia (HHT) are crucial. Furthermore, patients presenting with unexplained restrictive cardiomyopathy should be thoroughly evaluated for cardiac amyloidosis, especially when echocardiographic findings such as a granular, highly reflective myocardial texture and the characteristic cherry-on-top strain pattern are observed.

This case highlights the importance of heightened clinical awareness, timely diagnosis, and a collaborative, multidisciplinary approach in optimizing the management of complex cardiovascular diseases, particularly in resource-constrained settings.

## Conflict of Interest

The authors declare no conflicts of interest.

## Authors’ Contribution

**Conceptualization:** Naufal Fakhri Nugraha, Nabila Azka Namirah, Zahra Nadiah, Zahran Lazuardi Haryawan, and Abednego Panggabean

**Data Curation:** Zahra Nadiah, and Nabila Azka Namirah, Naufal Fakhri Nugraha

**Formal Analysis:** Nabila Azka Namirah**,** Naufal Fakhri Nugraha

**Investigation:** Nabila Azka Namirah, Naufal Fakhri Nugraha, Zahra Nadiah, Zahran Lazuardi Haryawan, and Abednego Panggabean

**Project Administration:** Nabila Azka Namirah

**Supervision:** Abednego Panggabean

**Validation:** Nabila Azka Naamirah, Naufal Fakhri Nugraha, Zahra Nadiah, Zahran Lazuardi Haryawan, and Abednego Panggabean

**Writing –Original Draft**: Nabila Azka Namirah, Naufal Fakhri Nugraha

**Writing –Review and Editing**: Nabila Azka Namirah, Naufal Fakhri Nugraha, Zahra Nadiah, Zahran Lazuardi Haryawan, and Abednego Panggabean

## Acknowledgement

We thank all staff at Pameungpeuk Hospital for their support in preparing this paper.

## Funding

This research has not received any specific grant from public, commercial, or non-profit sector agencies.

## Additional file

Video 1 : https://drive.google.com/drive/folders/1-U1FExoLBmryqacYJOhqcVDLvDAvtHKG?usp=sharing
